# Cross-Sectional Serological Survey for *Leptospira* spp. in Beef and Dairy Cattle in Two Districts in Uganda

**DOI:** 10.3390/ijerph14111421

**Published:** 2017-11-21

**Authors:** Anou Dreyfus, Terence Odoch, Lordrick Alinaitwe, Sabrina Rodriguez-Campos, Amanuel Tsegay, Valentine Jaquier, Clovice Kankya

**Affiliations:** 1 Section of Epidemiology, Vetsuisse Faculty, University of Zurich, 8006 Zürich, Switzerland; 2 College of Veterinary Medicine, Animal Resources & Biosecurity, Makerere University, P.O. Box 7062 Kampala, Uganda; odochterence@gmail.com (T.O.); lordricka@gmail.com (L.A.); eman96er@gmail.com (A.T.); clokankya@gmail.com (C.K.); 3 Institute of Veterinary Bacteriology, Vetsuisse Faculty, University of Bern, 3012 Bern, Switzerland; sabrina.rodriguez@vetsuisse.unibe.ch (S.R.-C.); valentine.jaquier@vetsuisse.unibe.ch (V.J.)

**Keywords:** leptospirosis, Microscopic Agglutination Test, Uganda, cattle, seroprevalence

## Abstract

Seroprevalence of *Leptospira* spp. in cattle is unknown in Uganda. The aim of this study was to estimate the seroprevalence of *L. interrogans* Icterohaemorrhagiae, Pomona, *L. kirschneri* Butembo, Grippotyphosa, *L. borgpetersenii* Nigeria, Hardjo, Wolfii, and Kenya and an overall seroprevalence in cattle from Kole and Mbale districts. Two hundred-seventy five bovine sera from 130 small holder farms from Kole (*n* = 159) and Mbale (*n* = 116), collected between January and July 2015, were tested for antibodies against eight *Leptospira* strains by Microscopic Agglutination Test. A titer of ≥100 was considered seropositive, indicating past exposure. Overall, the seroprevalence was 19.27% (95% CI 14.9–24.5%). Pomona seroprevalence was highest with 9.45% (6.4–13.7%), followed by Kenya 5.09% (2.9–8.6%), Nigeria 4.00% (2.1–7.2%), Wolfii 3.27% (1.6–6.3%), Butembo 1.86% (0.7–4.4%), Hardjo 1.45% (0.5–3.9%), and Icterohaemorragiae and Grippotyphosa with less than 1% positive. Seroprevalence did not differ between districts and gender (*p* ≥ 0.05). Seven animals had titers ≥400. Cross-reactions or exposure to ≥1 serovar was measured in 43% of serum samples. Seroprevalence of 19% implies exposure of cattle to leptospires.

## 1. Introduction

Leptospirosis is an important global bacterial zoonosis transmitted mainly by urine, either through direct contact with carrier animals or indirectly through contaminated water or soil. Transmission can also occur through aborted tissues [1]. *Leptospira* organisms are spirochaetes including 20 species, 25 serogroups (sg), and almost 300 serovars (sv). Serovars are adapted to mammalian hosts colonizing their renal tubules and are excreted in the environment for months or years [1,2,3].

The Leptospirosis Epidemiology Reference Group (LERG) of the World Health Organization (WHO) published a systematic literature review in 2015 estimating the worldwide annual leptospirosis incidence to be one million cases (95% CI, 305,000–1,750,000) with 58,900 deaths (95% CI, 23,800–95,800) because of leptospirosis. For East Africa, an annual incidence of 25.6 (95% CI 9.3–43.3) per 100,000 population was estimated [4].

The epidemiology of leptospirosis in Uganda has so far not been described thoroughly. The burden of leptospirosis in humans in Uganda is unknown. A serological survey found that 35% (126/359) of humans visiting two health centers in Western Uganda had antibodies against leptospiral serovars and 2% had antibody titers corresponding with probable recent leptospirosis when tested by the Microscopic Agglutination test (MAT). The most common serovar was *Leptospira (L.) borgpetersenii* sv Nigeria (sg Pyrogenes) with 19.8%, which had been isolated from bovines elsewhere in Africa [5]. Another prevalent sg in these patients was Sejroe represented by sv Hardjobovis and sv Wolffii with 5.6% and 5.3%, respectively. Individuals who had participated in skinning cattle recently had ten-fold higher odds of having antibodies to *Leptospira* compared to those who had not. A very low or nonexistent prevalence was found against sv Bratislava sg Australis with 1.9%, against sv & sg Grippotyphosa with 0.1%, and sv & sg Icterohemorrhagiae and sv Patoc sg Semaranga with 0.0%. Based on the prevalent serogroups and the risk factor analysis, a bovine–human transmission pathway was hypothesized [6].

The prevalence of leptospires in domestic and wild animal populations in Uganda, the economic impact of leptospirosis in livestock, and the importance of specific hosts transmitting and maintaining leptospires remain unknown. A few serological surveys in animals in Uganda have been published. A study in 105 dogs nearby three national parks resulted in 27% of dogs being seropositive against *L. interrogans* by the MAT, with Icterohaemorragiae (43%) and Canicola (39%) being the most frequent of six tested serovars. The other serovars were Pyrogenes (21.4%), Tarassovi (10.7%), and Grippothyposa and Australis (7.2% each) [7].

Atherstone et al. found in 2014 that 29% of tested cattle (*n* = 92) and 42% of buffaloes (*n* = 92) were seropositive against serovar Hardjo by ELISA [8]. However, this commercial IgM and IgG ELISA had not been validated for this geographical region and ecosystem, so the test sensitivity and specificity remain unknown. Studies on the seroprevalence of other serogroups/serovars in cattle in Uganda are missing.

In other East African countries (as defined by the United Nations Statistics Division in October 2013), the following serogroups have been identified from acute human leptospirosis cases (h) and animal carrier hosts (a): Australis (h & a)**,** Autumnalis (h & a), Ballum (a), Bataviae (a), Canicola (h & a), Grippotyphosa (h & a), Hebdomadis (h & a), Icterohaemorrhagiae (h & a), Mini (h), Pomona (a), Pyrogenes (h & a), Sejroe (a), and Tarassovi (h & a). Of these serogroups, cattle were described as potential carrier hosts for Australis, Hebdomadis, Bataviae, Pomona, Sejroe, Icterohaemorrhagiae, Pyrogenes, and Tarassovi [9].

This current study was conducted in Mbale and Kole districts in Uganda. Mbale is located in Eastern Uganda, 00°57’N, 34°20’E and the Kole district in the northern region of Uganda, 02°24’N, 32°48’E. The majority of household members in both districts are involved in agriculture as the main economic activities. In the Mbale district, about 57% of households and in Kole about 80% keep livestock, of which the majority are small holder farms [10]. The total cattle population was estimated at 63,826 for Mbale [11] and 22,000 for Kole (estimate from the district office).

The majority of farmers keep cattle together with other domestic animals including sheep, goats, dogs, domestic pigs, chicken, and rabbits. Cattle are generally left to graze freely around the homestead during the day; however, some farmers will keep them in a confined area or tether them. While chicken, sheep, and goats are left to free-range as well, pigs stay more in a constructed confinement. Animals usually drink from communal water sources including open wells and small streams, which will also at times be used by humans for domestic use and recreation. While in Kole wildlife–domestic animal interactions are very limited, they are comparatively more common in Mbale. In all of Uganda, vaccination of cattle is not commonly practiced against leptospirosis.

The rainy seasons in Mbale and Kole usually take place from March to June (1st) and from August to November (2nd), with the highest peak being around May and November. In Eastern Uganda, where Mbale is located, the mean annual rainfall varies from 1374 to 2058 mm depending on the area. In comparison, the average long-term annual rainfall for Uganda is 1318 mm [12]. In Kole, the average annual rainfall for 2015 was much lower with 123 mm [13]. Unfortunately, long-term data on rainfall patterns in Kole were not found in the literature. Farmers report an increasingly erratic rainfall pattern with more intense rainfall in the 2nd rainy season, causing flooding. While “La Niña” years tend to bring dry weather, “El Niño” years yield heavy rains [12].

The aim of this study was to estimate the seroprevalence of selected serogroups and serovars, as well as the overall seroprevalence in individual cattle in the Kole and Mbale districts in Uganda, and to describe the seroprevalence in cattle by sub-county and gender.

## 2. Materials and Methods

### 2.1. Study Area and Population

A cross-sectional seroprevalence survey was conducted in Kole in January 2015 and in Mbale district between June and July 2015 to collect blood samples from individual beef and dairy (dual purpose) cattle of small holder farmers (Figure S1). This study was undertaken in parallel with an epidemiological investigation of brucellosis in cattle, sheep, and goats (A. Tsegay et al., unpublished data). On consultation with the district veterinary offices, we purposively selected the two districts of Kole and Mbale based on previous history and reports of fever and abortions in cattle in the two districts. The units of interest were households and small holder farms keeping approximately 5–15 cattle. A multi-stage sampling strategy was used to select sub-counties and villages. In the Kole district, the households within villages were contacted by radio and asked to participate and bring their livestock to a common meeting place. In Mbale, households within villages were selected randomly from a list of households that were identified to keep cattle. This list was availed by the local veterinary authorities and the local council and one chairperson helped to identify the households. Cattle from each selected households were sampled based on consent sought from animal owners before sample collection. On average, two bovines were sampled per farm/household.

The samples were collected by Masters of Veterinary Preventive Medicine students and Ph.D. candidates at the College of Veterinary Medicine, Animal Resources and Biosecurity (COVAB), Makerere University in Kampala. The students were supervised by qualified staff in the field and from COVAB to ensure strict adherence to the protocol that was approved by the ethical review committee of COVAB. Seven to 10 mL of blood was drawn from the jugular or caudal vein of apparently healthy cattle using a 20 G needle, and divided between 4 mL plain (for serum) and 4 mL anticoagulant coated (for whole blood) vacutainers (Becton Dickinson BD^TM^). Information on age and gender of sampled bovines was acquired. All samples were left to clot at ambient temperature before serum was harvested into cryogenic tubes and maintained on ice until transportation to the Central Diagnostic Laboratory at COVAB for storage at −20 °C. Later the samples were couriered on dry ice for serological analysis at the Institute of Veterinary Bacteriology, University of Bern, Switzerland, where it was stored at −20 °C.

Scientific and ethical clearance to conduct this was obtained from the Uganda National Council of Science and Technology (UNCST).The study was reviewed by the research ethics committee and found to be scientifically and ethically satisfactorily and it was approved with a reference number A565.

### 2.2. Sample Size

The target sample size for collecting individual bovine blood samples was calculated with an estimated seroprevalence of 30%, based on the findings of Atherstone [8], a significance at *p* = 0.05, and a confidence level of 0.95, and was 323 bovines.

### 2.3. Serological Analysis

The presence of antibodies against pathogenic *Leptospira* was assessed by MAT according to OIE standards [14]. Live cultures of eight *Leptospira* spp. reference strains were used in this study (Table 1). Serogroups/serovars were chosen based on the occurrence in the previously described human study in Uganda [6] or when cattle are common carrier hosts.

In order to reduce infection risks, it is generally recommended to heat-inactivate serum samples for 30 min in a water bath at 56 °C prior to MAT testing [15]. However, in our study, the serum samples were heat-inactivated for 2 h using the protocol as applied by the Pirbright Institute to bovine sera to inactivate Foot and Mouth Disease Virus [16]. The effect of the heat-inactivation for 2 h on the performance of the MAT was assessed using two reference rabbit antisera against strain Hardjoprajitno sv Hardjo and strain RGA sv Icterohaemorrhagiae (Royal Tropical Institute, KIT Biomedical Research, Leptospirosis Reference Centre, Amsterdam, The Netherlands) as well as two positive bovine sera from the serum collection at the Institute of Veterinary Bacteriology (Vetsuisse Faculty, University of Bern, Bern, Switzerland).

The sera were initially screened at a dilution of 1:100. Those with a positive reaction were titrated in a serial two-fold dilution to determine the end-point titer defined as the reciprocal of the highest serum dilution at which ≥50% of the leptospires remain agglutinated. Sera with a titer of ≥100 were considered seropositive, i.e., indicating past exposure to leptospires [17].

### 2.4. Statistical Analysis

Data was recorded using Microsoft Excel 2010 (Microsoft Corp, Redmond, WA, USA) and analyzed with Stata 10 (StataCorp, College Station, TX, USA). Proportions of seropositive animals overall and for each serogroup and serovar listed in Table 1 and 95% confidence intervals [18] were calculated. Differences of seroprevalences by district, sub-county, and gender were described and analyzed by a chi square test. Serological titers and multiple exposure/cross-reactions were described by serogroup and serovar.

## 3. Results

### 3.1. Data Collection and Description of the Study Population

A total of 275 serum samples from 130 different herds/households were tested by MAT (Table 1). In Mbale, 465 bovines were sampled in the four sub-counties, Busiu, Busoba, Nakaloki, and Namanyoni. Due to limited funding, not all of these samples could be serologically tested and therefore 150 samples were randomly selected using Excel for testing at the Swiss national reference laboratory for leptospirosis. Of these, 116 samples were tested by MAT. In the Kole district, 187 bovines were sampled in the two sub-counties Aboke and Ayer and 159 tested by MAT.

The median age of the sampled animals was three years (range 1–15). Gender was equally distributed with 133 (48.4%) females and 142 (51.5%) males. Most animals were local breeds (Zebu or Ankole) (*n* = 266, 96.7%), six (2.2%) were cross (local-exotic) and three (1.1%) exotic (to Africa) breeds.

### 3.2. Serological Results

The use of the Pirbright protocol for heat inactivation has been shown to have little impact on subsequent serological testing [16], and no discrepancies between the MAT results before and after heat-inactivation were observed.

The number of seropositive animals (to one or more serovars) was 53/275 with an overall seroprevalence of 19.27% (95% CI 14.9–24.5%). Seroprevalence of sv Pomona was highest with 9.45% (6.4–13.7%), followed by Kenya with 5.09% (2.9–8.6), Nigeria with 4.00% (2.1–7.2), Wolfii with 3.27% (1.6–6.3), Butembo with 1.86% (0.7–4.4), Hardjo with 1.45% (0.5–3.9), and Icterohaemorragiae and Grippotyphosa with less than 1% positive (Table 2). The “overall seroprevalence” and the “Pomona seroprevalence” did not differ significantly in the two districts and by gender (*p*-value > 0.05). In Mbale, the overall (a) and Pomona (b) seroprevalence was 20.69% (a) and 12.93% (b), respectively, and in Kole 18.24% (a) and 6.92% (b), respectively. Among females, the overall (a) and Pomona (b) seroprevalence was (a) 20.30% and (b) 9.02%, respectively, and among males it was (a) 18.31% and (b) 9.86%, respectively. While the overall seroprevalence by sub-county was not statistically significantly different (*p*-value = 0.408), it was for Pomona seroprevalence (*p*-value = 0.033) (Table 3).

**Figure 1:**
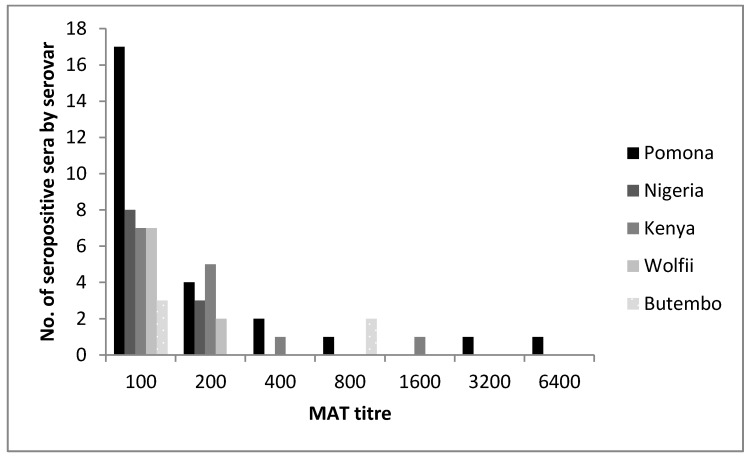
Frequency histogram showing the number of seropositive sera of cattle (*n* = 275) from Mbale and Kole districts in Uganda at each MAT titer to different *Leptospira* serovars or serogroups (one animal may be seropositive against several serovars).

The MAT titer range was 100–6400, with seven animals having titers ≥400 (Figure 1). Cross-reactions or exposure to ≥1 serovar was measured in 43% of serum samples. Frequently cross-reacting serovars were Pomona with Butembo, Kenya with Nigeria, and Hardjo with Wolfii (or vice versa, Table 4 and Table S1).

## 4. Discussion

This is to our knowledge the first report on the seroprevalence of various pathogenic *Leptospira* serogroups and serovars in cattle in Uganda. An overall seroprevalence of 19% implies exposure of cattle to leptospires, with sv Pomona making the largest contribution, followed by sv Kenya and Nigeria. Cattle are known to be maintenance hosts for sv Pomona [19] and possibly Nigeria [5] elsewhere. It is therefore possible that the seropositive cattle were not only exposed to leptospires in the past, but also function as maintenance hosts, likely exposing humans and other species by direct urine splashes or by shedding leptospires in water and the environment. The seroprevalence of Hardjo and Wolfii, typically associated with cattle in other places, was rather low in this study. In seroprevalence studies we can evaluate to a certain extent past exposure to leptospires. However, in order to understand the role of cattle as a maintenance host/carrier, the risk of shedding, and the veterinary public health relevance of bovine leptospirosis, the investigation of *Leptospira* prevalence in bovine kidneys and urine (shedding) by PCR is necessary. Therefore, we are currently conducting a cross-sectional study in two abattoirs in Kampala with the aforementioned objectives.

Pigs, wild boars, and other suid species are also known to be maintenance hosts for sv Pomona. Since many farmers keep cattle and pigs on the same farm, and contact with wild suid species may occur, the role of pigs in the *Leptospira* transmission cycle may be important and is worth exploring.

Given that communal water sources are a potential common source of infection and are shared between different animal species and humans, testing of water samples in areas with high leptospirosis prevalence and incidence may be useful.

In a human seroprevalence study, the most common serovar was sv Nigeria (sg pyogenes) with 19.8% [6]. Therefore, “One Health” research on the role of leptospires in undifferentiated (non-malarial) acute fever in humans and the investigation of risk factors for human leptospirosis in a case control study is highly recommended.

The measured seroprevalence in cattle of rodent associated serogroups/serovars, such as Icterohaemorrhagiae and Grippotyphosa was very low. It is known that sv Grippotyphosa does not induce a strong antibody response. Nevertheless, questions arise as to whether (a) rodents in these districts in Uganda play a minor role as carriers of *Leptospira* spp. or (b) in the transmission pathway with cattle and whether (c) rodents carry different serovars (i.e., sv Kenya?), or (d) the environmental contamination by rodents for an indirect transmission is in general low but may become high with inundations during heavy rainfall. Investigating rodent kidneys for *Leptospira* prevalence and sequencing the strains would shed light into the role of rodents in the leptospirosis transmission cycle in Uganda.

The seroprevalence was similar in both districts. This might be attributed to related farming ecosystems as well as cattle management systems in the two districts. In addition, there are informal reports that livestock movements occur across the Kole and Mbale districts. The Pomona seroprevalence difference by sub-county should be interpreted with caution, as the sample size of certain sub-counties was rather small.

The discrepancy between sampled and tested samples was due to insufficient serum volumes after serological testing for *Brucella*, evident hemolysis, and unclear labels due to illegible handwriting and rubbed off ink. The persons involved in sampling were informed about this to improve future studies. These reasons were random and should not have introduced a systematic selection bias. However, the study design and the sampling approach have limitations which may affect generalizability. First, the animals of the Kole district were a voluntary sample of farmers from randomly chosen villages, who brought their healthy animals to the sampling site. Whether farmers would rather take (a) sub-clinically ill cattle (which could suffer from leptospirosis), (b) healthy cattle, or (c) very healthy cattle, of which they were proud, is unknown. The first reason may slightly increase, and the third reason may decrease the *Leptospira* seroprevalence. In any case, since leptospirosis in cows mostly occurs asymptomatically in an endemic environment (at least when exposed to adapted serovars such as Pomona) [19] and we measured past exposure and not acute disease by MAT, the selection bias should be minimal.

We met the objective of determining the *Leptospira* seroprevalence in individual cattle in the Kole and Mbale districts for the tested serogroups/serovars, albeit with a slightly lower sample size (275 instead of 323). If we had chosen an apparent seroprevalence of 20% for our sample size calculation, 246 animals would have been sufficient for sample collection. Since we do not know the true prevalence, the exact required sample size remains unknown, but with 275 samples the power seems sufficient for a rather precise estimate.

However, the external validity of this study is limited given only two districts were targeted. To estimate a representative overall seroprevalence in cattle in Uganda, more districts would need to be enrolled. Further, not only the individual animal seroprevalence, but also herd seroprevalence should be estimated and risk factors for herd seropositivity investigated, while controlling for clustering. Moreover, information on clinical symptoms and abortions in cattle should be collected, in order to investigate an association between bovine leptospirosis and seropositivity.

The limited sampling period should not have affected the seroprevalence, as antibodies from natural exposure remain measurable for at least one year [20]. However, high titers may be more likely after the rainy seasons, which traditionally are March–June and August–November in Mbale and in Kole, which could potentially increase the exposure to leptospires.

Cross reactions occur frequently, especially between serovars of the same serogroup, such as Sejroe, but also in between serogroups; thus, determination of the infecting serovar by MAT is only presumptive [21]. Knowledge of cross reactions between serogroups/serovars may be helpful for future studies, where the MAT is used as diagnostic test.

Because of restricted funding, the size of the serovar panel was limited. The chosen panel was not large enough to cover all the common serogroups. For example, sg Australis had been identified in cattle in East Africa [9]. Moreover, the local strains are unknown, which would be a requirement for ideal serological testing. Hence, the overall prevalence may have been underestimated in this study and an important serogroup or serovar missed. Since testing was targeted towards past exposure to leptospires and not acute disease, an MAT sensitivity of 88% and specificity of 98% can be assumed [15]. Therefore, the tested prevalence is an “apparent seroprevalence” and will from this point of view be most likely slightly underestimated.

Given the limited knowledge on leptospirosis in Uganda, this study with pilot character is, despite its limitations, an initial stepping stone and delivers useful information for hypothesis generation and further leptospirosis research planning in Uganda.

## 5. Conclusions

An overall seroprevalence of 19% implies the exposure of cattle to leptospires. Cattle are maintenance hosts for sv Pomona and possibly sv Nigeria, likely exposing humans and other species by shedding leptospires at the human–livestock interface. Based on these findings, we initiated the investigation of *Leptospira* prevalence in bovine kidneys and urine by PCR in order to estimate the veterinary public health relevance of bovine leptospirosis in Uganda. Additionally, research on the role of leptospires in non-malarial fever in humans and the investigation of risk factors for human leptospirosis in a case–control study is highly recommended.

## Figures and Tables

**Table 1:** Strains of *Leptospira* spp. used as live antigens in the Microscopic Agglutination Test.

Genomospecies	Serogroup	Serovar	Strain
*L. interrogans*	Sejroe	Hardjo	Hardjoprajitno
	Sejroe	Wolfii	3705
	Icterohaemorrhagiae	Icterohaemorrhagiae	RGA
	Pomona	Pomona	Pomona
*L. kirschneri*	Autumnalis	Butembo	Butembo
	Grippotyphosa	Grippotyphosa	Moskva V
*L. borgpetersenii*	Ballum	Kenya	Njenga
	Pyrogenes	Nigeria	Vom

**Table 2:** Seroprevalence of *Leptospira* serovars and serogroups by Microscopic Agglutination Test (titer ≥1:100) among 275 bovines sampled in Kole and Mbale district, Uganda.

Serogroup	Serovar	n Positive ^1^	Prevalence ^2^ %	95% CI
Pomona	Pomona	26	9.45	6.4–13.7
Ballum	Kenya	14	5.09	2.9–8.6
Pyrogenes	Nigeria	11	4.00	2.1–7.2
Sejroe	Wolfii	9	3.27	1.6–6.3
Autumnalis	Butembo	5	1.82	0.7–4.4
Sejroe	Hardjo	4	1.45	0.5–3.9
Icterohaemorrhagiae	Icterohaemorrhagiae	2	0.73	0.1–2.9
Grippotyphosa	Grippotyphosa	1	0.36	0.0–2.3

^1^ Seropositive; ^2^ Seroprevalence. The overall seroprevalence was 19.27% (95% CI 14.9–24.5), with 53 animals being seropositive against one or more serovars.

**Table 3:** Frequency (N) and proportion (%) of sampled cattle by sub-county, and frequency (n) and proportion (%) of *Leptospira* spp. (“Lepto”) and Pomona seropositive cattle by sub-county of Mbale and Kole districts in Uganda.

District	Sub-County	*N* Sampled (%)	n Lepto ^2^ Positive (%)	n Pomona Positive (%)
Kole	Aboke	88 (32.0)	18 (20.4)	5 (5.7)
	Ayer	71 (25.8)	11 (15.5)	6 (8.4)
Mbale	Busoba	27 (9.8)	9 (33.3)	7 (25.9)
	Nakaloki	29 (10.5)	5 (17.2)	1 (3.4)
	Namanyoni ^1^	3 (1.1)	0 (0.0)	0 (0.0)
	Busiu	57 (20.7)	10 (17.5)	7 (12.3)
	Total	275 (100)	53 (19.3)	26 (9.4)

**^1^** Sample size too small for representative results; ^2^ seropositive against any tested serovar. While the overall seroprevalence was not statistically significantly different by sub-county (*p*-value = 0.408), the Pomona seroprevalence was (*p*-value = 0.033).

**Table 4:** Number and percentage of positive serum samples cross-reacting with other serovars and number of cross-reactions per serovar and serogroup.

Serogroup	Serovar	No of Positive Samples	No (%) of Cross-Reacting ^4^ Samples	No of Cross-Reactions ^5^	Main Cross-Reacting Serovar (No of Cross-Reactions)
Pomona	Pomona	26	7 (27)	12	Butembo (5)
Ballum	Kenya	14	5 (36)	10	Nigeria (4)
Pyrogenes	Nigeria	11	4 (36)	9	Kenya (4)
Sejroe	Wolfii	9	4 (44)	7	Hardjo (3)
Autumnalis	Butembo	5	5 (100)	7	Pomona (5)
Sejroe	Hardjo	4	4 (100)	4	Wolfii (3)
Ictero ^1^	Ictero ^1^	2	1 (50)	6	
Grippo ^2^	Grippo ^2^	1	1 (100)	6	
	Total	72 ^3^	31 (43)	61	

Multiple positivity of a serum against several serovars/serogroups could be due to cross-reactions against antigens in the MAT or because of multiple exposures to different serogroups/serovars. ^1^ Icterohaemorrhagiae; ^2^ Grippotyphosa; ^3^ 53 animals were seropositive against one or more serovars (=seroprevalence); however, because of cross-reactions, 72 positive MAT titers (≥100) against the listed serogroups/serovars were measured; ^4^ to one or more serovars; ^5^ one serum sample may cross-react with several serovars: each of these cross-reactions is summed up. The raw data of this table may be found in Table S1.

## Supplementary Materials

The following are available online at www.mdpi.com/1660-4601/14/11/1421/s1. Figure S1: Photo of sample collection site in Kole district, Table S1: Titers against eight leptospiral serovars of different serogroups assessed by Microscopic Agglutination Test in seropositive cattle from Mbale (*N* = 116) and Kole district (*N* = 159) in Uganda.
