# Supplementary material for: Cross-Sectional Serological Survey for Leptospira spp. in Beef and Dairy Cattle in Two Districts in Uganda
Source: Int J Environ Res Public Health. 2017 Nov 21;14(11):1421. doi: 10.3390/ijerph14111421 (PMC5708060; doi:10.3390/ijerph14111421)
Supplement: Supplementary file 1 [file ijerph-14-01421-s001.pdf]

International Journal of
Environmental Research and Public Health

MDPI

Supplementary Materials

Cross-Sectional Serological Survey for *Leptospira* spp. in Beef and Dairy Cattle in Two Districts in Uganda

Anou Dreyfus, Terence Odoch, Lordrick Alinaitwe, Sabrina Rodriguez-Campos, Amanuel Tsegay, Valentine Jaquier and Clovice Kankya

Table S1. Titers against eight leptospiral serovars of different serogroups assessed by Microscopic Agglutination Test in seropositive cattle from Mbale ($N = 116$) and Kole district ($N = 159$) in Uganda.

Sample ID	Grippo	Ictero	Hardjo	Butembo	Wolfii	Nigeria	Kenya	Pomona
**107**							100	100
**53**	100	100		100	100	100	200	100
**3**								100
**7**								100
**74**								100
**95**								100
**116**								100
**117**								100
**125**			100					100
**207**								100
**210**								100
**225**								100
**226**								100
**243**								100
**281**								100
**300**								100
**314**								100
**221**				100				200
**246**								200
**261**								200
**287**								200
**12**				100				400
**245**								400
**317**				800				800
**264**				800				3200
**35**								6400
**108**						100	100	
**123**						200	100	
**26**							100	
**57**							100	
**113**							100	
**198**							100	
**21**						200	200	
**183**							200	
**236**							200	
**242**							200	
**20**							400	
**307**							1600	
**270**					100	100		
**99**						100		
**120**						100		
**169**						100		

**182**				100
**267**				100
**320**				200
**54**			100	
**148**			100	
**170**			100	
**299**			100	
**318**		100	100	
**94**		100	200	
**192**		100	200	
**306**	100			

[a] Grippotyphosa; [b] Icterohaemorrhagiae.

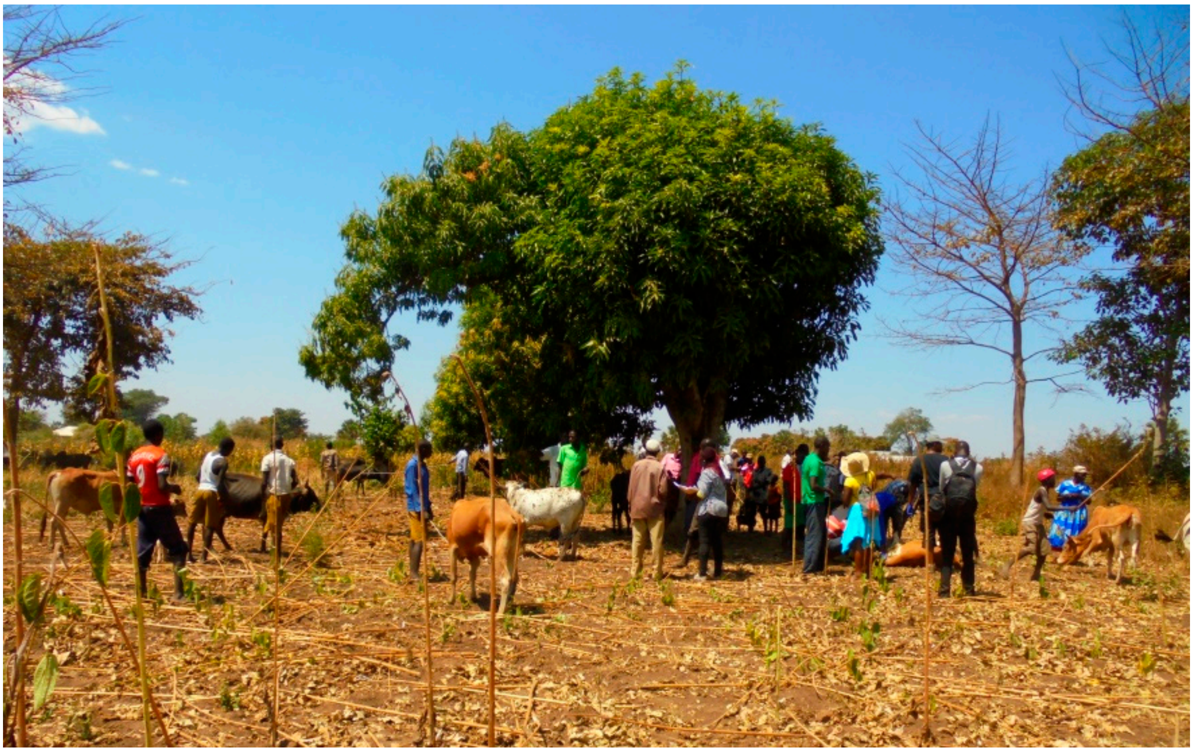

Figure S1. Sample collection site in Kole district.
